# Electrochemical Sensors Based on Screen-Printed Electrodes: The Use of Phthalocyanine Derivatives for Application in VFA Detection

**DOI:** 10.3390/bios6030046

**Published:** 2016-09-01

**Authors:** Amadou L. Ndiaye, Sébastien Delile, Jérôme Brunet, Christelle Varenne, Alain Pauly

**Affiliations:** 1 Clermont Université, Université Blaise Pascal, Institut Pascal, BP 10448, F-63000 Clermont-Ferrand, France; sebastiendelile@gmail.com (S.D.); brunet@lasmea.univ-bpclermont.fr (J.B.); christelle.VARENNE@lasmea.univ-bpclermont.fr (C.V.); Alain.PAULY@lasmea.univ-bpclermont.fr (A.P.); 2 CNRS, UMR 6602, Institut Pascal, Campus Universitaire des Cézeaux, 4 Avenue Blaise Pascal, 63178 Aubiere Cedex, France

**Keywords:** dropcast-deposition, metal-free phthalocyanine, acetic acid, CV, SWV

## Abstract

Here, we report on the use of electrochemical methods for the detection of volatiles fatty acids (VFAs), namely acetic acid. We used tetra-tert-butyl phthalocyanine (PcH_2_-tBu) as the sensing material and investigated its electroanalytical properties by means of cyclic voltammetry (CV) and square wave voltammetry (SWV). To realize the electrochemical sensing system, the PcH_2_-tBu has been dropcast-deposited on carbon (C) orgold (Au)screen-printed electrodes (SPEs) and characterized by cyclic voltammetry and scanning electron microscopy (SEM). The SEM analysis reveals that the PcH_2_-tBu forms mainly aggregates on the SPEs. The modified electrodes are used for the detection of acetic acid and present a linear current increase when the acetic acid concentration increases. The Cmodified electrode presents a limit of detection (LOD) of 25.77 mM in the range of 100 mM–400 mM, while the Aumodified electrode presents an LOD averaging 40.89 mM in the range of 50 mM–300 mM. When the experiment is realized in a buffered condition, theCmodified electrode presents a lower LOD, which averagesthe 7.76 mM. A pronounced signal decay attributed to an electrode alteration is observed in the case of the gold electrode. This electrode alteration severely affects the coating stability. This alteration is less perceptible in the case of the carbon electrode.

## 1. Introduction

The production and optimization of renewable energy to foil global warming have gained a huge interest. Among the renewable energies, biomass-derived fuels [1,2,3,4] represent potential serious candidates, which are able to gain energy just from fermentation. Such processes give rise to the production of volatile fatty acids (VFAs) [2,4]. Among the VFAs, acetic acid (AA) represents the most abundantly-produced VFA, during controlled fermentation processes. Therefore, its monitoring is a prerequisite for optimization. For example, in biogas production, the best working conditions for an efficient gas production yield are dependent on the AA concentration [5]. Therefore, to monitor the biogas production, the acetic acid concentration is of great importance. In fact, the VFAs should be produced in a certain concentration range to ensure better performances of the reactor and simultaneously keep the pH stable [6,7]. However, recent reports show that the microbial communities are able toresist changes in the VFA concentrations [7]. Current methods employed to control such process conventionally usecolorimetric and chromatographic techniques or the distillation process [8]. However, such methods require specific encumbering equipment and expertise, which arenot easy to implement.

These methods are well documented compared to electrochemical approaches, which are less explored. One reason explaining the lack of electrochemical methods dedicated to control such bio-processes is that thedirect oxidation or reduction of acetic acid is very difficult to achieve in conventional electrochemistry. The only existing methods approaching theelectrochemical methods are based on electrocatalytic processes. These destructive methods are based on catalytic reforming, which necessitated specific working conditions (high temperature, use of a catalyst, etc.) and suggest that the VFAs are completely transformed at the end [9,10,11,12,13]. All of these methods are still in use, but there is a necessity to develop new and easy to implement methods devoted to this research field.

Gaberkorn et al. [14] have studied the acid and base interaction of PcH_2_ in acidic solutions (with a higher concentration of acetic acid), and they showed that the protonation of the phthalocyanine occurring mainly on the meso-nitrogen can affect the highest occupied molecular orbital (HOMO)—lowest unoccupied molecular orbital (LUMO) band gap. In this study [14], the presence of the acetic acid appears to initiate the protonation. Moreover, Stuzhin et al. [15] described some phthalocyanine and porphyrin derivatives as multicenter conjugated systems with simultaneous acid and base properties. Following these observations, we have explored the ability of the phthalocyanines as a sensing material for acetic acid detection. Taking into account that we will use concentrations lower than 0.5 M, we can surely use such phthalocyanines without inducing dissociation.

Several phthalocyanines have been investigatedfor applications devoted to electrocatalysis [16,17,18] and also for sensing purposes [19,20,21]. The electrochemical properties of metal-free phthalocyaninesare conventionally attributed to ring redox processes, which are based on the gain or loss of electrons from their frontier orbitals [22]. Electrode modification employing phthalocyanines derivatives [23,24] has been already reported. Such a modification can, in principle, be achieved through electrodeposition [25,26,27], electropolymerization [20,28] and dropcasting [19]. The two first methods require specific conditions, such as the presence of anelectropolymerizable group (pyrrole, thiophene etc.) [20,28] or specific working conditions [26,27]; the latter presents the advantage of being easy to perform and accessible. In fact, in the dropcasting method, the preparation of the solution in an adequate solvent can allow one to perform electrode modification. Here, we explored the dropcasting methods on screen-printed electrodes (SPEs). However, the dropcast method presents some disadvantages: (i) low film stability; (ii) the rapid ageing effect; and (iii) the non-control of the layer thickness.

Here, we studied the electrode modification of a gold and carbon working electrode using a metal-free phthalocyanine. The deposition process is achieved by the dropcast deposition method, and the successful electrode modification will be revealed through characterization of the coating using CV techniques and scanning electron microscopy techniques. Finally, the electroanalytical performance of the PcH_2_-tBu-based modified electrodes towards acetic acid detection will be described by cyclic voltammetry experiments.

## 2. Materials and Methods

### 2.1. Materials and Reagents

All reagents were of analytical grade and purchased from Sigma-Aldrich. Dimethylformamide (DMF) and acetonitrile (ACN) were used as solvents without further purification. The sensing material consists of phthalocyanine derivatives, namely 2,9,16,23-tetra-tert-butyl-29H,31H-phthalocyanine (purity 97%), denoted as PcH_2_-tBu. Acetic acid (AA) was purchased from Fisher and used as the analyte. Tetrabutylammonium tetrafluoroborate (TBAB), potassium chloride (KCl; 3 M), potassium dihydrogen phosphate (KH_2_PO_4_ salt; >99%) andpotassium hydroxide (KOH; 1 M) were purchased from Sigma-Aldrichand used as electrolytes. Sulfuric acid (H_2_SO_4_; 0.5 M) was purchased from Fluka and used for the pretreatment of the electrodes.

### 2.2. Electrochemical Measurements

All measurements were carried out at room temperature. Electrochemical measurements were performed with a µSTAT 200 potentiostat (Dropsens, Oviedo, Spain) controlled by Dropview software. The electrodes consist of screen-printed electrodes (SPEs) (DRP C110 and DRP C220AT, Dropsens, Oviedo, Spain) [19,29]. The working (4 mm in diameter) and counter electrodes are made of carbon (C, DRP C110) or gold (Au, DRP C220AT), while the reference electrodes are made of silver (Ag). Such SPEs have been already used in related literature for electrode modification purposes [19,29,30]. All potentials are reported vs. the Ag pseudo-reference electrode. Cyclic voltammetry measurement were performed between −0.3 and +0.8 V for the gold electrodes and between −0.5 V and +1 V for the carbon electrodes. Square wave voltammetry wasconducted between −0.3 V and +0.4 V with a potential step of 10 mV; a potential pulse of 60 mV and at a frequency of 15 Hz.

### 2.3. Solutions’ Preparation

The solutions/dispersions are prepared by dissolving PcH_2_-tBu in a solvent mixture (DMF/ACN (1:5 volume ratio)) containing TBAB (0.1 M) as an electrolyte. This solvent mixture was chosen to dissolve the phthalocyanine. For the electrolyte choice, TBAB was preferred to KCl, since it dissolves in organic solvent, while adding KCl solution will lead to a two-phase solution mixture, which is not suitable for deposition. The final concentration of the stock solution is 0.5 mM. Stock solutions based on PcH_2_-tBu without electrolyte (TBAB) do not provide a significant electrochemical signal. The TBAB is used to enhance the conductivity in the coating (see Figure S1 in the Supporting Information). The buffered solutions (0.1 M) were prepared from KCl, KH_2_PO_4_ and 1 M KOH to obtain a final pH of 7.0.

### 2.4. Characterization of Modified Electrodes

For the characterization of the modified electrodes, cyclic voltammetry and scanning electron microscopy (SEM) were used.SEM micrographs are obtained from a Cambridge Scan 360 SEM operating at 3 kV. The samples were prepared by deposition of solution (dropcast) on the working electrodes. The coated electrodes were dried at 50 °C and were finally left overnight at room temperature.

### 2.5. Electrodes Modifications by Dropcasting

The deposition of the PcH_2_-tBu was performed on both gold and carbon working electrodes. Prior to the deposition, the electrodes were cleaned in 0.5 M H_2_SO_4_ by cycling the electrode potential from −0.1 V to +1 V (for the Au working electrode) and from−1 V to +1 V (for the C working electrode) with a scan rate of 0.1 V/s.The solutions for electrode modification prepared by mixing the PcH_2_tBu with solvent (see Section 2.3) were carefully deposited on the working electrodes. The coatings were dried at 50 °C and finally left overnight at room temperature.

### 2.6. Acetic Acid Detection Experiments

For the acetic acid (analyte) detection, KCl is used as the electrolyte. It is worth noting that TBAB can be also used as the electrolyte, but deliberately, we chose KCl for further development. In fact, KCl is used in the buffered solution [31,32] to analyze the VFA production, and in some other cases, KCl is also a component of the culture medium [33,34,35,36]. For the CV measurements, acetic acid solutions at concentrations between 0.001 M and 0.5 M containing 0.1 M KCl were prepared. To perform acetic acid detection experiments in buffered conditions, the same preparation procedure was used, except that the KCl was replaced by the buffered solution.

## 3. Result and Discussion

### 3.1. Electrode Modification by Dropcast Deposition

The dropcast deposition method is a very simple and sparing method. In fact, with a few µL of a solution, a coating can be realized. In a typical procedure of deposition, 1 µL ofthe stock solution (PcH_2_-tBu; 0.5 mM) was carefully dropped onto the working electrodes (C and Au) and allowed to dry at 50 °C for 1 h. Care must be taken to realize the drop cast with SPEs, since the counter eletrode (CE) and reference eletrode (RE) are in close contact with the working eletrode (WE). The CE and RE must be free of any deposit to ensure valuable electrochemical measurements. The electrodes were finally left overnight at room temperature to ensure complete solvent evaporation. The modified electrodes obtained by this procedure will be denoted ***C SPE-PcH_2_tBu*** and ***Au SPE-PcH_2_tBu*** in the following part of this article.

### 3.2. Characterization of the Modified Electrodes

**Figure 1:**
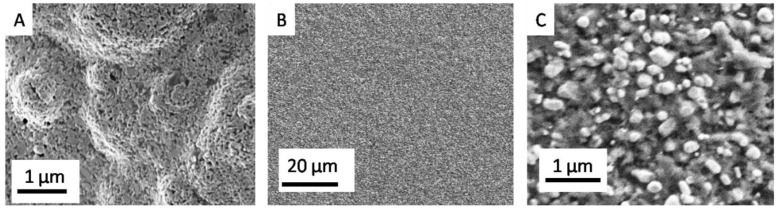
Representative SEM micrographs obtained before (**A**) and after dropcast deposition (**B,C**). (**C**) represents PcH_2_-tBumodified Au electrodes (**B**) at higher magnifications.

To highlight the effectiveness of the coating, we have performed SEM analysis of the modified electrodes and the uncoated Au SPE for comparison. The PcH_2_-tBu mixture has been carefully deposited on the working electrode, and the results are shown in Figure 1.

In the lower magnification, as displayed in Figure 1B, the PcH_2_-tBu appears as a relatively homogeneous layer. However, the higher magnification (Figure 1C) reveals the microstructure with a morphology close to aggregates or grain-like structures representing the PcH_2_-tBu. The aggregates are present in different sizes and forms. The formation of aggregates is instantaneous in this liquid phase since a sonication step prior to deposition does not alter the obtained morphology. The electrolyte is embedded in the sensing layer ensuring the conductivity. Such aggregate formation is a result of the stacking formation observed in the phthalocyanine molecules.

**Figure 2:**
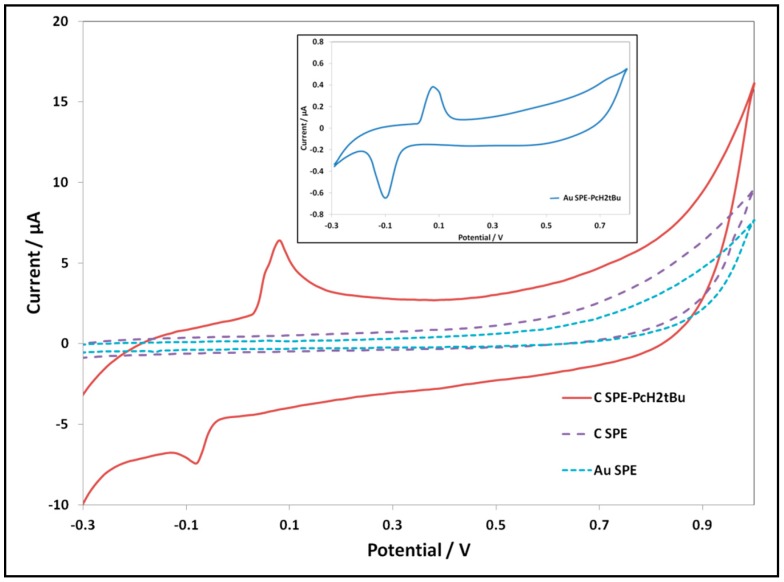
CV of the modified electrodes revealing the redox peaks of the PcH_2_tBu in KCl 0.1 M at a scan rate of 0.1 V/s. For comparison, the CV of the bare electrodes on the same KCl solution isshown. Inset: CV of Au SPE-PcH_2_tBu is represented in the inset, due to the low current intensity range.

The modified electrodes are also characterized by cyclic voltammetry. We performed cyclic voltammetry measurements of the modified electrodes in KCl (0.1 M) and compared them with CV of the bare electrodes. The results are shown in Figure 2. The ***Au SPE-PcH_2_tBu*** presents an oxidation peak at 0.08 V and its reduction at −0.1 V, while the ***C SPE-PcH_2_tBu*** shows an oxidation peak at 0.08 V and its reduction at −0.08 V.

Taking into consideration that bare electrodes do not present any defined peak within the electrochemical window, we can undoubtedly attribute these peaks as arising from the PcH_2_tBu. These peaks are assigned to phthalocyanine ring-based redox processes [24,37,38]. The ***C SPE-PcH_2_tBu*** modified electrode presents a quasi-reversible signal with a ΔEp(E_p,a_–E_p,c_) value of 160 mV, while the ***Au SPE-PcH_2_tBu*** shows a quasi-reversible signal with a ΔEp value of 180 mV. The I_p,a_/I_p,c_ ratios are 0.6 for ***Au SPE-PcH_2_tBu*** and 1.9 for ***C SPE-PcH_2_tBu***. For both ***C SPE-PcH_2_tBu*** and ***Au SPE-PcH_2_tBu*** modified electrodes, the ΔEp values are greater than 60 mV, and the I_p,a_/I_p,c_ ratios are different from one. Such behaviors suggest quasi-reversible processes. The deviation from reversibility (I_p,a_/I_p,c_ ≠ 1) in these phthalocyanine-based material compounds is presumably attributed to aggregation [24], but this deviation can also reveal the existence of chemical reactions following the electrochemical ones. The formation of aggregates given through the SEM analysis corroborates such results.

### 3.3. Acetic Acid Detection

**Figure 3:**
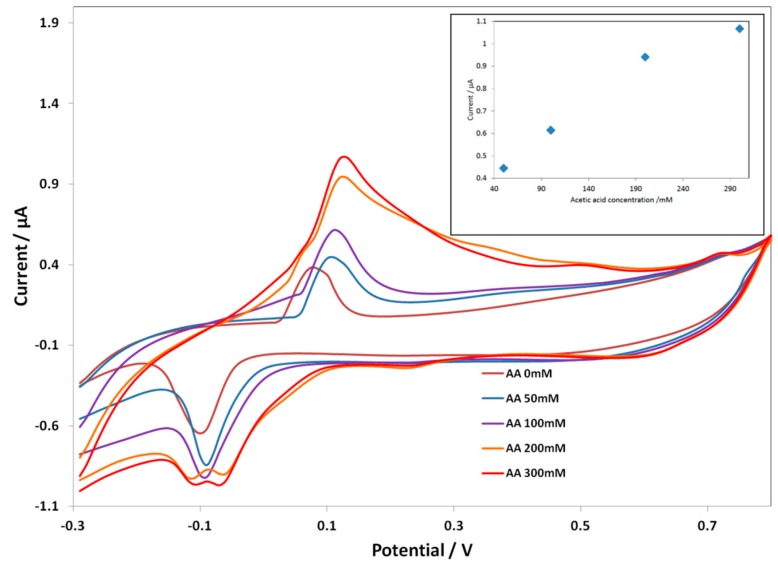
CV curves of Au screen-printed electrode(SPE)-PcH_2_tBu with the addition of the acetic acid in 0.1 M KCl. The CV labeled AA 0 mM represents the experiment in pure electrolyte (0.1 M KCl). Scan rate = 0.1 V/s.

Figure 3 shows the CV response of the ***Au SPE-PcH_2_tBu*** at different acid concentrations. At an acetic acid concentration of 50 mM, the ***Au SPE-PcH_2_tBu*** present peaks thatare localized at 0.11 V and −0.09 V. These peaksare slightly shifted compared to that obtained in pure electrolyte solution (represented by AA 0 mM in Figure 3).

By progressively increasing the amount of acetic acid, we observed a current increase as a function of the acid concentration. At a higher acid concentration, the oxidation peak is broadened, while the reduction peak seems to split into two peaks. This can be interpreted as resulting from an associated chemical reaction that occurs at a higher acid concentration. Moreover, up to 300 mM, the ***Au SPE-PcH_2_tBu*** electrode saturates. The broadening of the oxidation peaks combined with the occurrence of the splitting in the reduction side, at a higher acid concentration, can be associated with a chemical reaction occurring at a higher concentration and certainly leading to the modification of the structure. The calibration curve given in the inset of Figure 3 presents the linear dependence of the current intensity with concentration. From the calibration curve (inset in Figure 3), we can evaluate the limit of detection (LOD), which can be calculated following the 3 SD/m criterion [39], where m is the slope of the calibration graph and SD is the standard deviation of the voltammetric signal at the lowest concentration. We have calculate an LOD of 40.89 mM from the anodic peak at ca. +0.11 V.

**Figure 4:**
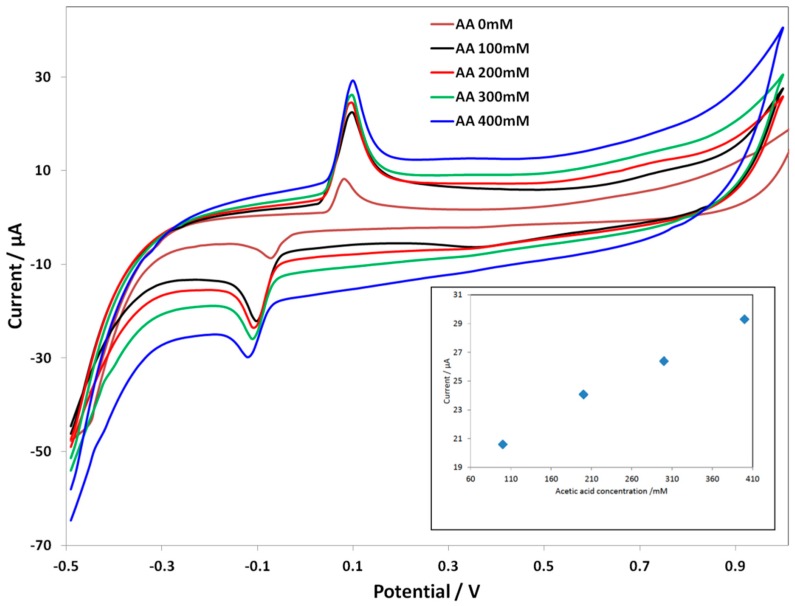
CV curves of C SPE-PcH_2_tBu with the addition of the acetic acid in 0.1 M KCl. The CV labeled AA 0 mM represents the experiment in pure electrolyte (0.1 M KCl). Scan rate = 0.1 V/s.

In the case of ***C SPE-PcH_2_tBu***, a different trend is observed. Figure 4 represents the CV of the modified electrode upon the addition of acetic acid. Here, the concentration range is from 100 mM–400 mM, since at a concentration lower than 100 mM, the signal is unclear. At an acetic acid concentration of 100 mM, the ***C SPE-PcH_2_tBu*** presents peaks thatare localized at 0.1 V and −0.1 V. These peaks are again shifted compared to those obtained in KCl. The calibration curve given in the inset of Figure 4 presents thelinear dependence of the current intensity with concentration. In the case of***C SPE-PcH_2_tBu***, the linear dependence is more accurate, as illustrated by the value of correlation coefficient R^2^ of 0.999. From the calibration curve, we have calculated an LOD of 25.77 mM from the anodic peak at ca. +0.10 V.

In both cases (***C SPE-PcH_2_tBu*** and ***Au SPE-PcH_2_tBu***), current versus concentration analysis revealed a linear dependence between the current peak and the analyte concentration. The different LOD values are attributed to the different electrocatalytic activity of the different electrodes. Even if this concentration range is currently high and the LOD in the mM range, these resultsshow that such modified electrodes can be used for acid acetic detection.

We have also evaluated the reproducibility and the lifetime of the electrochemical sensors. For this study, a solution containing 100 mM acetic acid has been used in successive experiments. A general current signal decreasing is shown, and this effect is more pronounced in the Au electrode. Indeed, the percentage of current decay is around 20%, based on 30 experiments, when gold is used as the working electrode, while this value averages 10% for the carbon electrodes. After several experiments, alsonew peaks coming probably from the Au electrodes’ oxidation are identified for the ***Au SPE-PcH_2_tBu***. In summary, after 40–50 experiments, the sensors show a general alteration, which is characterized by a pronounced flattening of the current peaks. The lifetime has been set to 50 CV cycles. This effect is more accelerated when higher acetic acid concentrations are used. Therefore,the lifetime of 50 CV is only valid if we work inthe above-mentioned conditions. The deposit seems to be more efficient on the carbon electrode compared to the gold electrode. Taking into account that the carbon electrode contains graphitic layers, we can explain this behavior by the possibility of the PcH_2_tBu to interact with the carbon electrode via the π−π interaction in a manner similar to the non-covalent interaction between carbon nanotubes and phthalocyanine derivatives [40].

**Figure 5:**
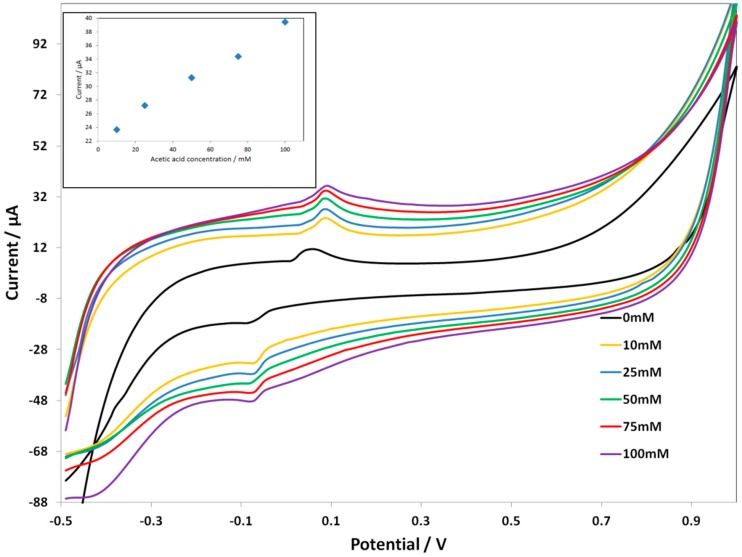
CV curves of C SPE-PcH_2_tBu with the addition of the acetic acid in 0.1 M buffer solution. The CV labeled AA 0 mM represents the experiment in pure electrolyte. Scan rate = 0.1 V/s.

**Figure 6:**
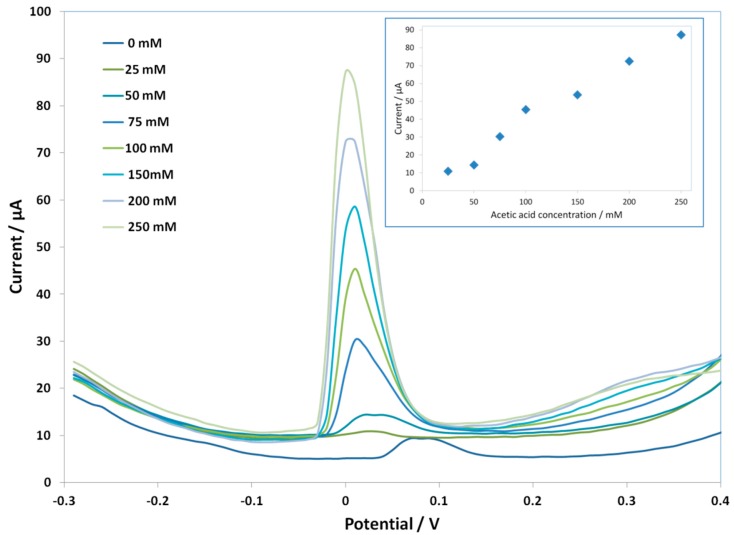
SWV curves of C SPE-PcH_2_tBu with the addition of the acetic acid in 0.1 M buffer solution. Potential step = 10 mV; potential pulse = 60 mV; frequency = 15 Hz. The SWV curve labeled as 0 mM represents the experiment in pure electrolyte. The insert shows the current dependencewith the acetic acid concentration.

Since the Cmodified electrodes were more stable, we performed an experiment with the ***C SPE-PcH_2_tBu*** to check the electroanalytical performance in buffered solutions by both the CV and SWV techniques. To this end, acetic acid has been added to a 0.1 M buffered solution (see the experimental part) to reach a concentration between 10 mM and 300 mM. The results are presented in Figure 5 (for the CV) and Figure 6 (for SWV). At the acetic acid concentration of 10 mM, the ***C SPE-PcH_2_tBu*** presents peaks thatare localized at +0.09 V and −0.08 V. We can notice that, in the buffered condition, the lowest attainable concentration giving an electrochemical signal is 10 mM by CV, while this value attains 25 mM by SWV. However, a broadening of the CV peaks occurs in buffered media compared to the peaks obtained in unbuffered media. This is explained by the high background current, which is exalted in the buffered media.

The sensor displays a linear response to acetic acid in the range of 10 mM–100 mM (inset in Figure 5) with a correlation coefficient R^2^ of 0.99. We obtained an LOD of 7.76 mM from the anodic peak at ca. +0.09 V. This result shows that the modified electrodes can be used in buffered, as well as in unbuffered media;aresult thatwill be useful for further development in bioreactors.

We have also performed SWV experiment on the ***C SPE-PcH_2_tBu*** for the acetic acid detection. The SWV parameters have been optimized (see the caption of Figure 6) and used for recording the SWV current signal. The SWV responses are displayed in Figure 6, and the insert contains the SWV current evolution as a function of the acetic acid concentration. We depicted, from these results, a current increase upon increasing the acid concentration. The SWV results are concomitant with those observed in the acetic acid detection using CV techniques. The responses are clearly identified, but the increase in the signal is still low. Nevertheless, we can clearly depict a shift between only KCl solution and acetic acid-containing solutions. This shift is more clearly evidenced than in the CV methods.

At this stage, it would be interesting to compare the electroanalytical performance of our modified electrodes to other electrochemical system in terms of the sensing of acetic acid. However, in front of the poor number of articles or reports dealing with acid acetic detection using electrochemical methods, we are unable to provide a valuable comparison.

**Figure 7:**
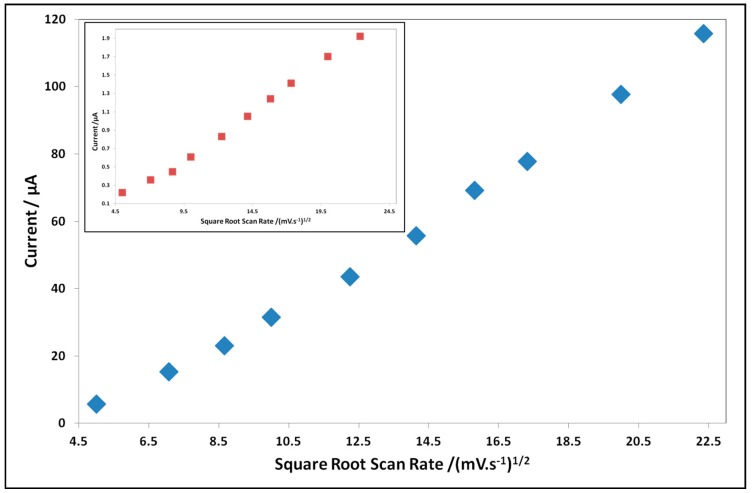
The evolution of current peak of the modified electrodes upon increasing the scan rate in 100 mM acetic acidsolution. Due to the difference in the current range, the case of ***Au SPE-PcH_2_tBu*** is displayed in the inset.

After performing the evaluation of the sensing performance, we have also conducted sensing experiments on the modified electrodes to characterize the redox process taking place in such modified electrodes. Therefore, we recorded the evolution of the current peak while increasing the scan rate of the modified electrodes in the presence of the analyte. This study has been conducted in a 100 mM acetic acid solution containing 100 mM KCl with a scan rate from 25–500 mV/s. Figure 7 shows the evolution of the anodic current peak as a function of the increasing scan rate.

An increasing potential scan rate gives rise to an increase of the current peak as shown in Figure 7 for all modified electrodes. The ***PcH_2_tBu*** modified electrodes show a linear dependence of the anodic peak current with the square root of the scan rate. The linearity dependence is also confirmed by the regression coefficient (R^2^) value (R^2^ = 0.994 for ***C SPE-PcH_2_tBu*** and R^2^ = 0.995 for ***Au SPE-PcH_2_tBu***) and is indicative of a predominant diffusion-controlled redox process. The difference in slopes is attributed to the difference in the nature of the electrode material.

Taking into account that the potential peaks in phthalocyanine and porphyrin derivatives are attributed to the electron’s promotion from the HOMO or LUMO energy levels, the presence of the acid will induced a perturbation on the delocalized pi-electrons (through protonation or coordination), which will be traduced into a change on the HOMO/LUMO band gap positioning. In fact, the spectroscopic studies by Gaberkorn et al. [14] in the case of the PcH_2_ have revealed that protonation induces a change in the HOMO/LUMO band gap positioning, causing a shift in UV-visible spectra. In the protonated or unprotonated form, the PcH_2_tBu can also interact with the acetate anion, which is a highly coordinating ligand. Taking into account the previous studies about phthalocyanines and the acid base interaction [14,15,41], we have proposed a two-step mechanism: (i) a first step leading to a protonation; (ii) and the subsequent coordination of the analyte (acetate form or acetic form). However, the fact that protonation induces a change can be regarded as a drawback since other inorganic acids (different from VFA) will also induce a similar change, and this effect will point to a lack of selectivity. However, taking into account that acetic acid is the most abundantly-produced in bioreactors, its detection can be useful to control the monitoring of the whole VFA production.Even if the protonation is the first step in the mechanism, the coordination of the carboxyl and carboxylate group to the macrocycle center containing a delocalized π-system is responsible for the response observed. This behavior is confirmed by the responses recorded in buffered media by both CV and SWV experiments. 

## 4. Conclusions

Here, we investigated the possibility to modifySPEs with metal-free phthalocyanine for acetic acid detection by the dropcast method. The electrode modification processed through dropcast deposition is a simple and sparing method, which presents serious potential for further development in the field acetic acid detection. Theircharacterization by cyclic voltammetry and SEM analysis, as well as the evaluation of their electroanalytical performances have shown the effectiveness of such electrodes’ modification and their potential use for electrochemical detection. The carbon electrodes present a high background current, but allow to obtain stable electrodes. On the contrary, the gold electrode, which seems to be well suited for electroanalytical experiments, presents some limits (oxidation, stability). We showed here that these modified electrodes can be used to detect acetic acid. The detected concentration is currently limited in the mM range, but we intend to improve the process and to lower the detection limit in further investigation. This work opens a new window on the utilization of macrocycle-based material for acid acetic detection and proposesnew ideas for VFA detection.

## Supplementary Materials

Supplementary File 1
