# Supplementary material for: Electrochemical Sensors Based on Screen-Printed Electrodes: The Use of Phthalocyanine Derivatives for Application in VFA Detection
Source: Biosensors (Basel). 2016 Sep 1;6(3):46. doi: 10.3390/bios6030046 (PMC5039665; doi:10.3390/bios6030046)
Supplement: Supplementary File 1 [file biosensors-06-00046-s001.pdf]

Supplementary Materials: Electrochemical Sensors Based on Screen-Printed Electrodes: The Use of Phthalocyanine Derivatives for Application in VFA Detection

Amadou L. Ndiaye, Sebastien Delile, Jérôme Brunet, Christelle Varenne and Alain Pauly

This figure shows the CV response of the modified electrodes obtained from the PcH2-tBu stock solutions with TBAB electrolyte (blue curve) and from stock solutions without TBAB electrolyte (green curve). We can see that the modified electrode in which TBAB is embedded in the coating presents a more intense signal. This is the reason why we decided to add TBAB into the PcH2tBu solution.

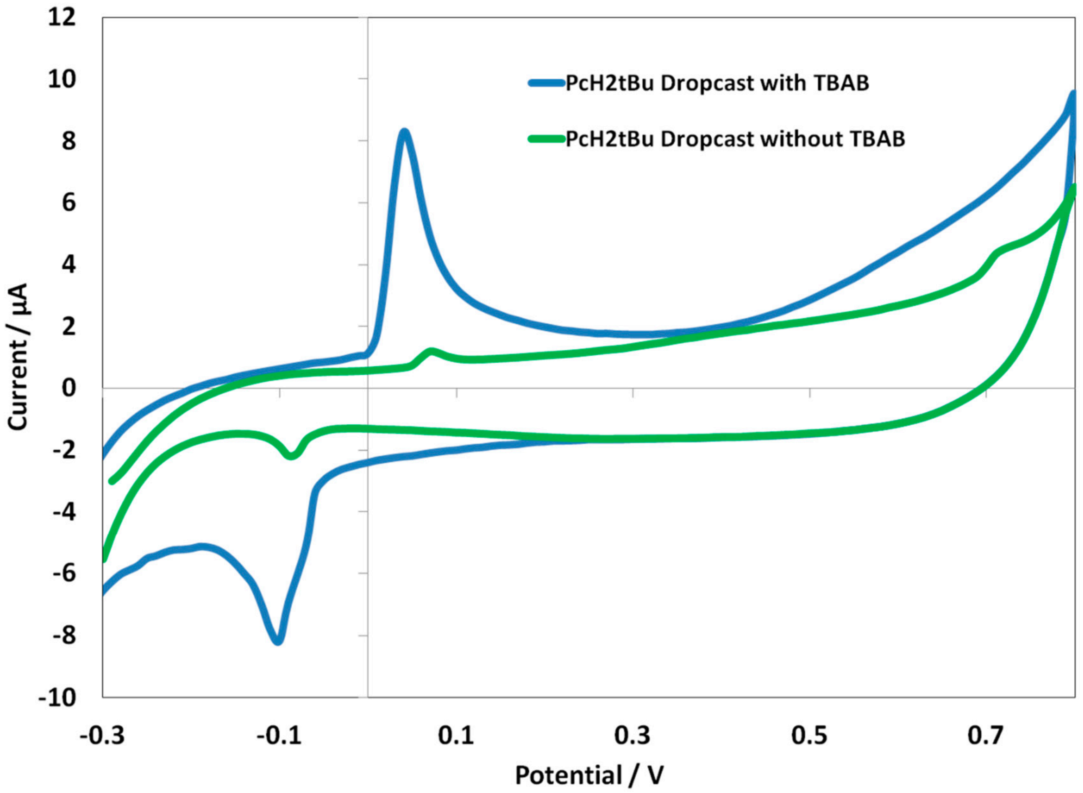

Figure S1. Example of CV curves obtained after electrode modification with TBAB and without TBAB. The CVs are obtained in 0.1 M KCl solution. Scan rate 0.1 V/s.
